# The impact of digital literacy on university students' innovation capability: evidence from Ningbo, China

**DOI:** 10.3389/fpsyg.2025.1548817

**Published:** 2025-07-08

**Authors:** Xingyu Zhou, Kaili Sun, Kai Zhu, Linru Feng, Qi Sun, Dongdong Zhong

**Affiliations:** ^1^Communist Youth League, Ningbo University, Ningbo, China; ^2^School of Business, Ningbo University, Ningbo, China; ^3^Ningbo University of Finance and Economics, Ningbo, China; ^4^School of Civil & Environmental Engineering and Geography Science, Ningbo University, Ningbo, China

**Keywords:** digital literacy, digital competence, innovation capability, higher education, university students

## Abstract

**Introduction:**

Digital literacy has become a necessary basic ability for university students and has profoundly influenced their learning attitudes and behavior. This study aims to explore the direct effects of digital literacy on innovation capability among university students and whether it varies with students' personal characteristics.

**Methods:**

This study developed new scales to measure the digital literacy and innovation capability of university students. Data was collected from 12 universities in Ningbo, China (*N* = 1,334) through a self-report questionnaire and analyzed using Structural Equation Modeling (SEM).

**Results:**

The findings showed that digital literacy positively correlated with university students' innovation capability (β = 0.76, *p* < 0.001). Significant differences in digital literacy and innovation capability were found among university students with different educational backgrounds, academic disciplines, types of institutions, and training experiences, but no gender differences were observed.

**Discussion:**

University students with better digital literacy tend to be more innovative. The study suggests that higher education institutions should emerge from the traditional concept of curriculum systems, accelerate the construction of a digital literacy cultivation ecosystem considering different personal characteristics, and stimulate the innovation drive of university students.

## 1 Introduction

As emphasized by the United Nations Development Programme (United Nations Development Programme, 2021), the fostering of innovation is of paramount importance for accelerating solutions to development challenges and shaping a sustainable, equitable, and inclusive future. Innovation is a vital process across industries and represents a significant source of competitive advantage for businesses (Tweneboah-Koduah et al., 2020). A revised European Union (EU) agenda for higher education institutions (European Commission, 2017) emphasizes the distinctive role of higher education in fostering innovation. A significant number of educational institutions are seeking methods to cultivate students' innovative capabilities. However, the majority of these approaches are based on traditional perspectives of knowledge transmission and educators, such as the design of innovative learning environments (Richardson and Mishra, 2018; Ovbiagbonhia et al., 2019; Phi and Clausen, 2021), utilization of theory-derived instructional interventions (Mayhew et al., 2021), and establishment of a culture of innovation (Selznick et al., 2021). Although education plays a pivotal role in fostering human innovation skills, some studies indicate that higher education institutions are not the sole arbiters of such needs (Badcock et al., 2010; Quintana et al., 2016). Consequently, it is imperative to investigate how to stimulate university students' intrinsic motivation for innovation from the learners' perspective.

In the digital age, the continuous advancement of digital technologies, including the Internet, big data, and artificial intelligence (AI), has rendered digital literacy a significant ability for university students to adapt to the times, affecting their knowledge acquisition and lifelong learning. Digital technologies have become integral to the ways in which people interact and communicate. Digital technologies are deeply embedded in human interaction and communication, transforming individual learning behaviors (Thompson, 2016; Masanet et al., 2019) and contributing to the cultivation of students' creativity in specific ways (Glăveanu et al., 2019; Fielding and Murcia, 2022; Liu et al., 2022; Quah and Ng, 2022). Students with higher digital literacy possess strong data organization and analysis skills, enabling efficient learning and effective information integration (Cui, 2017). This facilitates superior academic performance (Tadesse et al., 2017; Mehrvarz et al., 2021) and enhances career adaptability (Zhou et al., 2023). These findings suggest that university students with higher digital literacy may be more innovative. However, as this is a relatively new area of research, there are currently limited in-depth investigations of this topic in different educational contexts.

This study aimed to evaluate the digital literacy and innovation capabilities of university students using a proper model and investigate the relationship between the two. In particular, this study addresses the following three questions: Q1. Does the improvement of university students' digital literacy enhance their innovation capability? Q2. Which areas of digital literacy are more closely related to innovation capability? Q3. What demographic factors affect university students' digital literacy and innovation capability?

As one of the fastest-growing developing countries, China has recently placed significant emphasis on fostering students' innovative capabilities. The 2024–2035 master plan on building China into a leading country in education, which was jointly issued by the Communist Party of China Central Committee and the State Council, clearly proposed “to improve the mechanism for discovering and cultivating outstanding innovative talents.” Prior to this, the Ministry of Education, in conjunction with 11 central units and local governments, created the China College Students' “Internet +” Innovation and Entrepreneurship Competition. This competition has been held for 10 consecutive years, becoming an important platform for promoting innovation and entrepreneurship education reform in colleges and universities, as well as an international event where college students realize their dreams of innovation and entrepreneurship. As one of the most rapidly developing regions in China in terms of digital technology and the digital economy, Ningbo has considerable experience implementing digital transformation in education. This makes it an ideal case study for exploring the relationship between digital literacy and innovation capability from the learner's perspective. Therefore, this study empirically investigates this relationship, fills the gaps in related topics, and provides new evidence and insights into the field of innovation in higher education.

We present a scale for measuring the digital literacy and innovation capability of university students, developed with consideration of the educational and cultural context in China. The scale was validated through a questionnaire survey of university students from 12 universities and colleges in Ningbo, China. This study employs correlation analysis and structural equation modeling (SEM) to empirically analyze the relationship between students' digital literacy and innovation capability, identify the relatively important dimensions of digital literacy for innovation, and explore the differences in digital literacy and innovation capabilities among different groups of university students. In conclusion, the findings of the research are discussed and summarized, and recommendations are made regarding the enhancement of innovation abilities through the utilization of digital literacy.

The rest of this paper is structured as follows: Section 2 presents a systematic review of the literature and the research questions posed. Section 3 outlines the research methods employed in this study, including the selection of the sample, assessment tools used, and analysis methods applied. Section 4 presents the research findings and provides responses to the research questions. Section 5 discusses the empirical results of the data. Section 6 presents the conclusions drawn from the analysis and suggestions for further research.

## 2 Literature review

### 2.1 Definition of digital literacy

The term “digital literacy” was initially introduced by Gilster (1997) to refer to “the ability to understand and use information in multiple formats from a wide range of sources when it is presented via computers.” Over time, the academic community has expanded the definition of digital literacy beyond the mere functional use of technology and adaptation of skills to include composite dimensions such as cognitive and attitudinal dimensions (Martin, 2006; Bawden and Robinson, 2009; Tang and Chaw, 2016).

In light of the accelerated advancement and pervasive integration of digital technology, critical thinking, security awareness, and a sense of responsibility have been incorporated into digital literacy (Chan et al., 2017; Ng, 2012; Brown et al., 2020). In particular, with the rapid iteration and upgrade of generative AI (Gen-AI) represented by ChatGPT, ethical issues such as data abuse and evasion of responsibility have become increasingly prominent, driving the transformation of digital literacy into a comprehensive capability system. Therefore, Gen-AI is now included as an important part of students' digital literacy (Bender, 2024).

The terms digital competence and digital literacy are often considered synonymous. However, the term digital literacy is more frequently used in higher education research. Regional differences in the usage of these terms indicate that digital literacy is widely used in the UK, the US, and Asia, while the definition of digital competence is mostly derived from the EU and South America (Spante et al., 2018).

### 2.2 How to measure digital literacy

Eshet-Alkalai (2004) was the first to propose a comprehensive digital literacy framework, comprising five dimensions: photo-visual, reproduction, branching, information, and socio-emotional. Subsequently, Eshet-Alkalai (2012) proposed the addition of real-time thinking in response to the demands of the digital era. Since the advent of the twenty-first century, several countries and international organizations have published digital literacy frameworks in response to the accelerating pace of digitization. The most significant frameworks have been developed by the EU and the United Nations Educational, Scientific and Cultural Organization (UNESCO). Since 2013, the EU has published four iterations of the Digital Competence Framework for Citizens (DigComp). The most recent iteration of the framework, DigComp 2.2, retains the five dimensions that were initially proposed. The five domains are information and data literacy, communication and collaboration, digital content creation, safety, and problem-solving (Vuorikari et al., 2022). In 2015, the UK's Joint Information Systems Committee (JISC) issued the Digital Abilities Framework (Beetham, 2015), which is highly similar to DigComp but more finely categorized. In 2018, UNESCO, utilizing DigComp 2.0 as a preliminary point of reference, incorporated two additional dimensions, namely “Devices and software operations” and “Career-related competences,” with the objective of proposing a Digital Literacy Global Framework (DLGF) for learners (Law et al., 2018). In 2022, the Ministry of Education of the People's Republic of China published “Digital Literacy for Teachers” (Ministry of Education of the People's Republic of China, 2020), which underscored the significance of digital reflection in relation to digital activities, thereby emphasizing the importance of digital awareness. Table 1 provides a detailed summary of these frameworks.

**Table 1:** Summary of internationally recognized digital literacy frameworks.

**Publisher (source)**	**Covering dimensions**	**Applicable groups**
EU (Vuorikari et al., 2022)	Information and data literacy, communication and collaboration, digital content creation, safety, problem solving	All citizens
UK^a^ (Beetham, 2015)	ICT proficiency, information, data and media literacies, digital creation, problem solving and innovation, digital communication, collaboration and participation, digital learning and development, digital identity and wellbeing	Staff in any role and students in any educational setting
UNESCO (Law et al., 2018)	Information and data literacy, communication and collaboration, digital content creation, safety, problem solving, devices and software operations, career-related competences	Digital literacy seekers and learners
China^b^ (2022)	Digital awareness, digital knowledge and skills, digital application, digital social responsibility, professional development	Teachers

^a^Beetham (2015).

^b^Ministry of Education of the People's Republic of China (2020).

Research on digital literacy frameworks for non-student populations has progressed. However, no internationally authoritative digital literacy framework has been specifically designed for university students. Some scholars have synthesized existing authoritative digital literacy frameworks to develop frameworks and assessment scales for university students' digital literacy. Monteiro and Leite (2021) proposed a scale for identifying university students' digital literacy based on the digital literacy framework by Martin and Grudziecki (2006) and DigComp 2.0. (Fang and He, 2022), based on DigComp 2.1, incorporated students' learning and career development abilities into the assessment, leading to a revised university students' digital literacy scale that covers five dimensions including, information and data, communication and collaboration, content and creation, digital and life, and learning and development. Both scales refer to DigComp, and the latter incorporates the individual characteristics of university students. However, both fail to acknowledge the crucial role of digital awareness in cultivating digital literacy.

In a recent study, Li et al. (2023) developed a digital literacy framework tailored to the needs of Chinese students. To create this framework, the researchers conducted a comprehensive comparison and analysis of several internationally authoritative digital literacy frameworks. Their framework encompasses four dimensions: technological practice, higher-order thinking and competence, cognitive-emotional and literacy, and socio-cultural, which are specifically divided into 11 subdimensions. While the sociocultural dimension of this framework is oriented toward the macro level and may not be directly applicable to the micro-level individual, the overall framework effectively balances commonality and individuality, offering valuable theoretical insights for developing a digital literacy framework tailored to Chinese university students.

The literature review revealed certain commonalities between digital literacy frameworks for university students and assessment models for other social groups. It can be argued that university students, as part of the general population, are a group receiving higher education and are therefore the teaching audience for educators. In theory, a digital literacy framework for university students should reflect the commonality of digital literacy for citizens, accommodate the individuality of learners' digital literacy, and align with the cultivation goals of educators' digital literacy. Accordingly, this study aims to construct a novel digital literacy evaluation model, beginning with the extant public digital literacy evaluation framework and integrating the distinctive attributes of Chinese university students.

### 2.3 Definition of innovation capability

As with the measurement of any capability, the measurement of innovation capability requires the integration of knowledge, skills and attitudes (Ovbiagbonhia et al., 2019). As an essential component of personal competence, innovation capability can be viewed as a set of abilities built from various competencies or skills, including but not limited to creativity, critical thinking, problem discovery and resolution, perseverance, and diverse communication and collaboration abilities (Hero et al., 2017; Keinänen et al., 2018; Pérez-Peñalver et al., 2012). Creativity is regarded as a significant component of innovation capability (Hurt et al., 1977; Chell and Athayde, 2011). It manifests as the ability to create innovative solutions by expanding, connecting, and reorganizing ideas when encountering certain problems or fields (Antonietti, 2011).

### 2.4 How to measure innovation capability

At the time of writing, there is no consensus on an indicator system for measuring university students' innovation capability. Building on the three-dimensional innovation capability model (individual, interpersonal, and networking) proposed by Penttilä and Kairisto-Mertanen (2012), Watts et al. (2012) developed a new three-dimensional innovation capability assessment scale through expert judgment and a literature review. Subsequently, Marín-García et al. (2013) re-validated this innovation capability assessment scale (Watts et al., 2012) and concluded that the scale generally met the standards of content validity and validation testing for forming a formal model. The three dimensions were interpreted as follows.

(1) The individual scale identifies an individual's capabilities regarding various innovation processes within an organizational context. These include target-oriented and tenacious actions, independent thought and decision-making, problem-solving and the development of working methods, persistence, risk-taking, and personal outlook. (2) The interpersonal scale is based on the assessment of communication, teamwork, and team leadership abilities. (3) The network scale encompasses the capability to establish and sustain productive relationships, collaborate effectively in a diverse and interdisciplinary setting, and communicate and interact effectively in an international context.

Subsequently, Keinänen et al. (2018) employed the aforementioned three-dimensional innovation capability assessment tool to conduct exploratory factor analysis on 495 questionnaires completed by students at four Finnish universities. This resulted in the development of a five-dimensional model instrument, comprising creative problem-solving, systems thinking, goal orientation, teamwork, and networking competencies. This refined framework is consistent with the individual-interpersonal-networking structure and has both theoretical and practical significance. In subsequent empirical studies, the five dimensions of this framework were also referred to as creativity, critical thinking, initiative, teamwork, and networking (Keinänen and Kairisto-Mertanen, 2019).

Furthermore, the external environment is regarded as a crucial factor influencing innovation capability (Chan and Yuen, 2014). In a pioneering contribution to the field, Amabile et al. (1996) were the first to include the environment as a component of creativity from the perspective of ability composition. The external environment that influences innovation capability can be broadly classified into theoretical and practical aspects. The theoretical environment encompasses several factors, including the classroom atmosphere, the specific domain content being learned (Keinänen and Oksanen, 2017), the presence of multidisciplinary learning environments, flexible curriculum settings, and internationalized environments (Keinänen and Kairisto-Mertanen, 2019). The practical environment encompasses both work undertaken within and beyond the classroom and school setting (Davies et al., 2013; Ovbiagbonhia et al., 2019) and the integration of research, development, and innovation (RDI) activities (Keinänen and Kairisto-Mertanen, 2019).

### 2.5 The relations of digital literacy and innovation capability

In the digital age, the acquisition of learning resources, transformation of learning methods, and innovation of learning environments are inextricably linked to digital technology. Students with high digital literacy can access a plethora of learning resources, utilize online learning platforms for self-improvement, manage knowledge, and showcase learning outcomes through digital tools with ease. Furthermore, digital technology provides a plethora of online opportunities in the realms of entertainment, communication, information and education (Rodríguez-de-Dios et al., 2018). The utilization of digital technology by university students facilitates interdisciplinary research and practice, thereby enhancing their innovative thinking and problem-solving abilities.

Nevertheless, there is a paucity of literature examining the influence of digital literacy on innovation capability from the student's perspective. The extant literature tends to concentrate on the influence of digital literacy on students' learning attitudes and behaviors (Getenet et al., 2024; He and Zhu, 2017; Prior et al., 2016). In the field of economics, some scholars have emphasized the significance of digital competence for organizations in promoting innovative work behavior through systematic literature reviews (Huu, 2023). However, the existing literature provides some insights. This study will analyze the impact of digital literacy on university students' innovation capability, focusing on creativity, critical thinking, initiative, and problem-solving skills.

#### 2.5.1 Digital literacy and creativity

Rashid and Rahman (2014) conducted a study comprising 5 professional interior architects and 10 interior design students at a community college in Malaysia. The utilization of social networking sites, such as Facebook, for online mentoring activities was found to facilitate collaboration between learners and professionals, thereby enhancing the creativity of learners. Bereczki and Kárpáti (2021) reported the results of interviews with educators and students, which indicated that activities involving the use of digital technology and the creation of digital art facilitate students' imaginative conjectures, explorations, and expressions, thereby enhancing creative thinking. These activities mainly include the utilization of authentic data collection and analysis instruments in student-driven scientific investigations, the deployment of content creation tools to experiment with diverse concepts in art and language arts activities, and the integration of interactive media and digital games in social studies. In a systematic review of the literature, Tang et al. (2022) identified several digital technologies that have been shown to enhance students' creativity. These include information preservation and sharing, digital games, digital design, digital writing, robotics, and virtual learning environments. The use of these technologies has been found to increase motivation, professional activities, higher-order thinking, creative collaboration, and cognitive load. These findings support the idea that digital technology can contribute to improving students' creativity and that students with higher digital literacy are better equipped to navigate digital technologies.

#### 2.5.2 Digital literacy and critical thinking

In a study conducted by Fu (2013), a list of the benefits and opportunities of using information and communication technology (ICT) was compiled. It was found that the use of ICT helps students effectively and efficiently acquire digital information and course content, supports student-centered and autonomous learning, and develops creative learning environments, thereby providing more opportunities for critical thinking skills. The utilization of digital technologies for collaborative discussion and information retrieval and sharing on social media can facilitate students' engagement with the subject matter in a more interactive and critical manner (Smith and Storrs, 2023). The creation of digital games empowers students to act as digital game designers and authors, thereby fostering the development of critical thinking skills (Carolyn Yang and Chang, 2013).

#### 2.5.3 Digital literacy and initiative

In accordance with the tenets of social cognitive theory, initiative can be defined as an individual's capability to proactively engage in their own learning process and take the initiative in determining their own actions to achieve the desired learning outcomes (Bandura, 1997). Students may be discouraged from taking the initiative to learn if they hold negative attitudes and possess limited knowledge of digital technology. Nevertheless, research indicates that during the COVID-19 pandemic, higher digital literacy enhanced online learning initiatives and helped reduce students' technical pressure and burnout (Kumpikaite-Valiuniene et al., 2021).

#### 2.5.4 Digital literacy and problem-solving skills

Digital games can simulate a variety of real-life problems, thereby providing students with authentic and relevant contexts for problem-solving (Annetta, 2008) and assisting them in developing a more nuanced understanding of the causal relationships between decision-making behaviors and outcomes (Ebner and Holzinger, 2007). Consequently, game-based learning methodologies have been demonstrated to be highly effective in enhancing students' problem-solving abilities (Yang, 2012; Tangkui and Keong, 2020; Cheng et al., 2023). Furthermore, artificial intelligence (AI) is transforming human interaction with machines. For example, ChatGPT, an advanced AI-powered natural language processing tool, has the potential to transcend disciplinary boundaries (Chaka, 2023), thereby empowering students to address complex problems more autonomously and effectively.

## 3 Methods

### 3.1 Sample and data collection

As the digital economy and innovation vitality of Ningbo are at the forefront of China, it has achieved remarkable results in promoting the digital transformation and development of colleges and universities, covering different types and levels of universities and colleges, which meets the needs of this research sample. The survey was conducted between March and April 2024 and targeted students from 12 universities and colleges in Ningbo, China. We used a simple random sampling (SRS) method and an online questionnaire to obtain data, and a total of 1,454 electronic questionnaires were distributed. Following the exclusion of invalid responses due to selective and patterned answering, 1,334 valid questionnaires were finally collected, resulting in an effective response rate of 91.75%. The disciplines were classified according to the International Standard Classification of Education (United Nations Educational, Scientific and Cultural Organization, 2011). (ISCED) published by the United Nations Educational, Scientific and Cultural Organization (UNESCO) in 2011, except for those related to the service industry. The institutions were classified according to their type, resulting in the following distribution: one flagship university, three general universities, two independent colleges, and six vocational colleges. We also asked participants if they had attended courses, lectures, or training sessions related to digital literacy (e.g., big data, blockchain, AI, Python, MATLAB). The demographic characteristics of the sample are shown in Table 2.

**Table 2:** Demographic characteristics of the participants.

**Variable**	**Category**	***N***	**Percent (%)**
Gender	Male	555	41.60
	Female	779	58.40
Education level	Junior college (Age range: 18–22 years)	551	41.30
	Undergraduate (Age range: 22–25 years)	783	58.70
Discipline	Engineering, manufacturing and construction	252	18.89
	Humanities and arts	210	15.74
	Science	126	9.45
	Education	38	2.85
	Social sciences, business and law	344	25.79
	Health and welfare	13	0.97
	Agriculture	77	5.77
	Others	274	20.54
Type of institution	Flagship university	158	11.84
	General university	374	28.04
	Independent college	255	19.12
	Vocational college	547	41.00
Training experience	Yes	760	56.97
	No	574	43.03

### 3.2 Scale design

A review of the literature in Section 2 reveals that some researchers have developed specific digital literacy and innovation capability scales for university students. However, these scales may not be entirely compatible with the actual circumstances of students in Ningbo, China. Accordingly, based on previous studies (Vuorikari et al., 2022; Li et al., 2023; Marín-García et al., 2013; Keinänen and Kairisto-Mertanen, 2019), this study retains some typical dimensions with strong applicability while making innovative adjustments and modifications in some of the dimension divisions and specific descriptions to better align with the characteristics of the survey respondents. To guarantee the quality of the questionnaires, a preliminary study was conducted before the formal survey. Based on the findings of this pilot study, the questionnaire items were modified to ensure reliability and validity of the instrument. The scales employed in the formal survey, along with the results of the reliability and validity tests, are presented below a detailed list of the questionnaire items used in the study is provided in Appendix 1.

In terms of testing reliability and validity, this study used internal consistency reliability (Cronbach's alpha) and composite reliability (CR). Cronbach's alpha and CR values >0.7 were used as the criteria for assessing the reliability of the model. Besides, multiple goodness-of-fit indexes were used to evaluate the applicability of the models, including the chi-square to degrees of freedom ratio (χ^2^/*df* , reflecting the similarity between the sample covariance matrix and the estimated variance matrix), the Root Mean Square Error of Approximation (RMSEA, the square root of the average squared residuals between predicted and actual values divided by the sample size), the Standardized Root Mean Square Residual (SRMR, the standardized form of the mean and standard deviation of residuals), the Comparative Fit Index (CFI, measuring the difference between predicted and actual values), the Goodness-of-Fit Index (GFI, testing whether the data distribution in the sample is reasonable), and the Tucker-Lewis Index (TLI, assessing the proportion of the difference between the current model and the initial model relative to the difference between the ideal model and the initial model). The χ^2^/*df* < 5, RMSEA and SRMR < 0.08, GFI, CFI, and NFI > 0.90 were taken as the criteria for a good model fit to the data (Schumacker and Lomax, 2004; Byrne, 2001; McDonald and Ho, 2002; Hu and Bentler, 1999; Bentler, 1982).

#### 3.2.1 Digital literacy scale

This study makes reference to the comprehensive, scientific and localized digital literacy framework proposed by Li et al. (2023), with the original dimension of “digital culture” excluded and the consideration of “digital awareness” included as the foundation of digital literacy. Ultimately, through the synthesis of prior research, a new digital literacy framework was defined and delineated, culminating in the formulation of a novel digital literacy scale for university students, comprising four dimensions and 14 sub-dimensions, as illustrated in Table 3.

**Table 3:** Digital literacy scale for university students.

**Dimensions**	**Sub-dimensions**
A. Digital awareness (DA)	A1. Digital self-awareness
	A2. Digital global awareness
	A3. Digital learning willingness
B. Digital technology practice (DTP)	B1. Information retrieval ability
	B2. Basic digital technology practice
	B3. Advanced digital technology practice
C. Higher-order thinking and ability (HTA)	C1. Digital communication and collaboration
	C2. Digital information sharing
	C3. Problem-solving abilities
	C4. Digital innovation
D. Cognitive emotion and responsibility literacy (CERL)	D1. Data literacy
	D2. Digital health and safety
	D3. Digital responsibility and accountability
	D4. Lifelong learning and sustainable development

Digital Awareness (DA) can be defined as the proactive mental reflection of digital-related activities and is an essential foundation for cultivating digital literacy. It comprises micro and macro digital cognition and willingness to learn digital technology resources. Digital Technology Practice (DTP) describes the ontological requirements for a world in which technology is ubiquitous. It encompasses the use of digital technology and the creation of digital works. This dimension is subdivided into three sub-dimensions: information retrieval ability, basic digital technology practice, and advanced digital technology practice. Higher-order Thinking and Ability (HTA) refer to the production activities that exceed mechanical repetition, including digital communication and collaboration, digital information sharing, problem-solving abilities, and digital innovation, which are further divided into four subdimensions. Cognitive Emotion and Responsibility Literacy (CERL) represents a pivotal aspect that extends beyond intellectual factors in human development in the digital age, encompassing abilities that digital intelligence cannot reach. This dimension comprises four sub-dimensions: data literacy, digital health and safety, digital responsibility and accountability, and lifelong learning and sustainable development.

Students demonstrate proficiency in assessing their digital literacy (Holm, 2024). Accordingly, a 5-point Likert scale was employed for self-assessment, with responses ranging from 1 (strongly disagree) to 5 (strongly agree). The Cronbach's alphas of the overall digital literacy scale and its four sub-scales were 0.94, 0.81, 0.88, 0.90, and 0.86, respectively. The CR were 0.97, 0.81, 0.89, 0.91, and 0.87, indicating good reliability. The results of the confirmatory factor analysis (CFA) demonstrated satisfactory structural validity, with all fit indices falling within the acceptable range as, χ^2^/*df* = 4.39, RMSEA = 0.06, SRMR = 0.03, CFI = 0.98, GFI = 0.96, and TLI = 0.97.

#### 3.2.2 Innovation capability scale

It is widely recognized that capabilities can be categorized into realistic capabilities and potential capabilities based on whether they have been acquired or not (Mirza et al., 2022; Embretson and Reise, 2000; Samejima, 1997). Similarly, based on previous research on innovation capability (Marín-García et al., 2013; Keinänen et al., 2018; Keinänen and Kairisto-Mertanen, 2019), this study divided innovation capability into two dimensions: Realistic Innovation Capability (RIC) and Potential Innovation Capability (PIC). Combining the characteristics of the assessed subjects, a new innovative ability scale for university students containing 12 subdimensions was constructed, as shown in Table 4.

**Table 4:** Innovation capability scale for university students.

**Dimensions**	**Sub-dimensions**
Realistic innovation capability (RIC)	Q1. Problem-identification ability
	Q2. Critical thinking
	Q3. Creativity
	Q4. Self-directed learning
	Q5. Networking
	Q6. Teamwork
Potential innovation capability (PIC)	R1. Courage
	R2. Curiosity
	R3. Initiative
	R4. Insight
	R5. Theoretical environment
	R6. Practical environment

RIC refers to the innovation skills developed or demonstrated by an individual. These skills can be grouped into six sub-dimensions: problem-identification ability, critical thinking, creativity, self-directed learning, networking, and teamwork. PIC refers to innovation skills that are not yet evident but could be developed through appropriate learning and practice. It includes four personal qualities: courage, curiosity, initiative, and insight, as well as two learning environment factors: the theoretical and practical environments (Davies et al., 2013; Keinänen and Kairisto-Mertanen, 2019; Keinänen and Oksanen, 2017; Ovbiagbonhia et al., 2019).

Similarly, a 5-point Likert scale was used for scoring, with responses ranging from 1 (strongly disagree) to 5 (strongly agree). The Cronbach's alpha of the overall innovation capability scale and its two sub-scales were 0.94, 0.90, and 0.88, respectively, while the CR was 0.95, 0.91, and 0.90, indicating good reliability. The results of the confirmatory factor analysis (CFA) demonstrated satisfactory structural validity, with all fit indices falling within the acceptable range: χ^2^/*df* = 4.78, RMSEA = 0.06, SRMR = 0.04, CFI = 0.95, GFI = 0.90, and TLI = 0.94.

### 3.3 Data analysis

The data was initially tested by Common Method Bias (CMB) using the IBM SPSS Statistics version 27 software. Then, descriptive statistics were employed to analyze the overall scores of university students in digital literacy (comprising DA, DTP, HTA, and CERL) and innovation capability (encompassing RIC and PIC). A correlation analysis was conducted on these variables. Subsequently, Structural Equation Modeling (SEM) was employed using AMOS 26 software to analyze the structural relationships between variables, with the objective of identifying which fields of digital literacy are more closely related to innovation capability. Finally, SPSS 27 was employed to investigate the demographic factors that influence university students' digital literacy and innovation capability.

This study evaluates the model fit using χ^2^/*df* , RMSEA, SRMR, GFI, CFI, and TLI (detailed in Section 3.2.1). Mean (M) and Standard Deviation (SD) were employed to assess the central tendency and differences in the data. A higher M value indicates a higher average, whereas a higher SD value indicates a greater deviation from the mean. Additionally, in the correlation analysis, the Correlation Coefficient (r) was used to measure the strength and direction of the linear relationship between two variables. The r value ranges from [−1, 1], with values closer to 1 indicating a strong positive correlation, values closer to −1 indicating a strong negative correlation, and values near 0 indicating no correlation between the two variables. The probability value (*p*) was used to assess the statistical significance of the linear relationship between the two variables, with *p* < 0.05 indicating a significant linear relationship and *p* ≥ 0.05 indicating a non-significant relationship. In the SEM analysis, the β coefficient represents the estimated change in the dependent variable for a one-unit change in the predictor variable. The *p* coefficient measures the significance of the standardized factor loadings between observed variables and their corresponding latent variables, with *p* < 0.001 used as the criterion in this study to indicate that the selected observed variables effectively reflected the latent variables. For the heterogeneity analysis, this study applied an independent-sample *t*-test and one-way ANOVA. The former compares the mean differences between two independent groups to determine whether there is significant heterogeneity, whereas the latter explores whether there are significant differences in means across multiple groups.

## 4 Results

### 4.1 Common method bias test

To minimize potential confusion and misleading conclusions caused by self-report biases, it is common practice to test for common method bias prior to analyzing the data. As illustrated in Table 5, the single-factor model exhibited a poor fit, with a χ^2^/*df* ratio of 22.07, RMSEA of 0.13 (>0.08), SRMR of 0.08, GFI of 0.59 (< 0.90), CFI of 0.75 (< 0.90), and TLI of 0.73 (< 0.90). These results indicate that common method bias is not a significant issue. In contrast, the six-factor model demonstrated a superior fit, with a χ^2^/*df* ratio of 4.81, RMSEA of 0.05, SRMR of 0.03, and GFI, CFI, and TLI statistics exceeding the reference standard of 0.9. These results indicate that the six-factor model outperformed the other models. Therefore, it can be concluded that no significant common method bias was present in this study. Although we have ruled out the serious confusion that may be caused by artificially introduced biases to the research results, it is helpful to consider using behavioral measures or qualitative interviews to deeply understand the subjective experience, behavioral motivation, and complexity behind social phenomena in future research.

**Table 5:** Results of the common method bias test.

**Model**	**Factor combination**	**χ^2^/*df***	**RMSEA**	**SRMR**	**GFI**	**CFI**	**TLI**
Six factors	DA, DP, HT, CR, RI, PI	4.81	0.05	0.03	0.92	0.96	0.94
Five factors	DA, DTP, HTA, CERL, RIC+PIC	6.04	0.06	0.03	0.90	0.94	0.94
Four factors	DA, DTP, HTA+CERL, RIC+PIC	8.52	0.08	0.04	0.85	0.91	0.90
Three factors	DA, DTP+HTA+CERL, RIC+PIC	10.24	0.08	0.05	0.82	0.89	0.88
Two factors	DA+DTP+HTA+CERL, RIC+PIC	11.57	0.09	0.05	0.80	0.88	0.86
Single factor	DA+DTP+HTA+CERL+RIC+PIC	22.07	0.13	0.08	0.59	0.75	0.73
Reference standards		<5	<0.08	<0.08	>0.90	>0.90	>0.90

DA, digital awareness; DTP, digital technology practice; HTA, high-level thinking and ability; CERL, cognitive emotion and responsibility literacy; RIC, realistic innovation capability; PIC, potential innovation capability; “+” indicates factor merging. For models other than single factor, only the best fitting model is listed.

### 4.2 Descriptive statistics and correlation analysis

Following the validation of all scales, this study proceeded to conduct descriptive statistics and correlation analysis on the various dimensions of university students' digital literacy and innovation capability. The results are presented in Table 6.

**Table 6:** Descriptive statistics and correlation analysis results.

**Variable**	***M***	***SD***	**1**	**2**	**3**	**4**	**5**	**6**
1. DA	3.49	0.68	1					
2. DTP	3.50	0.67	0.79^***^	1				
3. HTA	3.59	0.64	0.73^***^	0.85^***^	1			
4. CERL	3.72	0.62	0.65^***^	0.72^***^	0.80^***^	1		
5. RIC	3.67	0.58	0.62^***^	0.64^***^	0.67^***^	0.74^***^	1	
6. PIC	3.63	0.57	0.62^***^	0.59^***^	0.65^***^	0.73^***^	0.94^***^	1

^***^Correlation coefficient (r) is significant at the 0.001 level (two-tailed).

First, regarding the dimensions of digital literacy, the mean scores for DA, DTP, HTA, and CERL were 3.49, 3.50, 3.59, and 3.72, respectively, with standard deviations of 0.68, 0.67, 0.64, and 0.62. This indicates that the level of CERL was the highest; HTA among students was relatively lower, and improvement was required regarding their DA and DTP. The differences observed between the samples across these dimensions were relatively small. Second, with regard to the dimensions of innovation capability, university students displayed greater RIC (*M* = 3.67, *SD* = 0.58) than PIC (*M* = 3.63, *SD* = 0.57).

Correlation analysis indicated significant positive correlations among all factors. This suggests that improvements in one dimension of digital literacy (DL) may be accompanied by enhancements in other dimensions. Specifically, CERL had a stronger positive correlation with RIC (*r* = 0.74, *p* < 0.001) and PIC (*r* = 0.73, *p* < 0.001) than with other dimensions of DL. This suggests that competencies such as data literacy, digital health and safety, digital responsibility and accountability, and lifelong learning and sustainable development are particularly important in shaping individuals' digital innovation and collaboration capabilities. From a practical perspective, this means that policies and training programs aimed at improving these aspects of digital literacy may have greater benefits in fostering innovation and teamwork capabilities than focusing solely on technical skills.

Furthermore, compared to the other dimensions, DTP exhibited a weaker positive correlation with RIC (*r* = 0.64, *p* < 0.001) and PIC (*r* = 0.59, *p* < 0.001), suggesting that while digital practical skills are still positively correlated with innovation and collaboration, their influence is slightly weaker. This highlights the importance of improving digital literacy as a whole, as technical skills alone may not fully drive innovation outcomes without the support of broader digital capabilities. These findings highlight the multifaceted nature of digital literacy and its role in improving key aspects of personal capability in the digital age.

### 4.3 Structural equation modeling analysis

**Figure 1:**
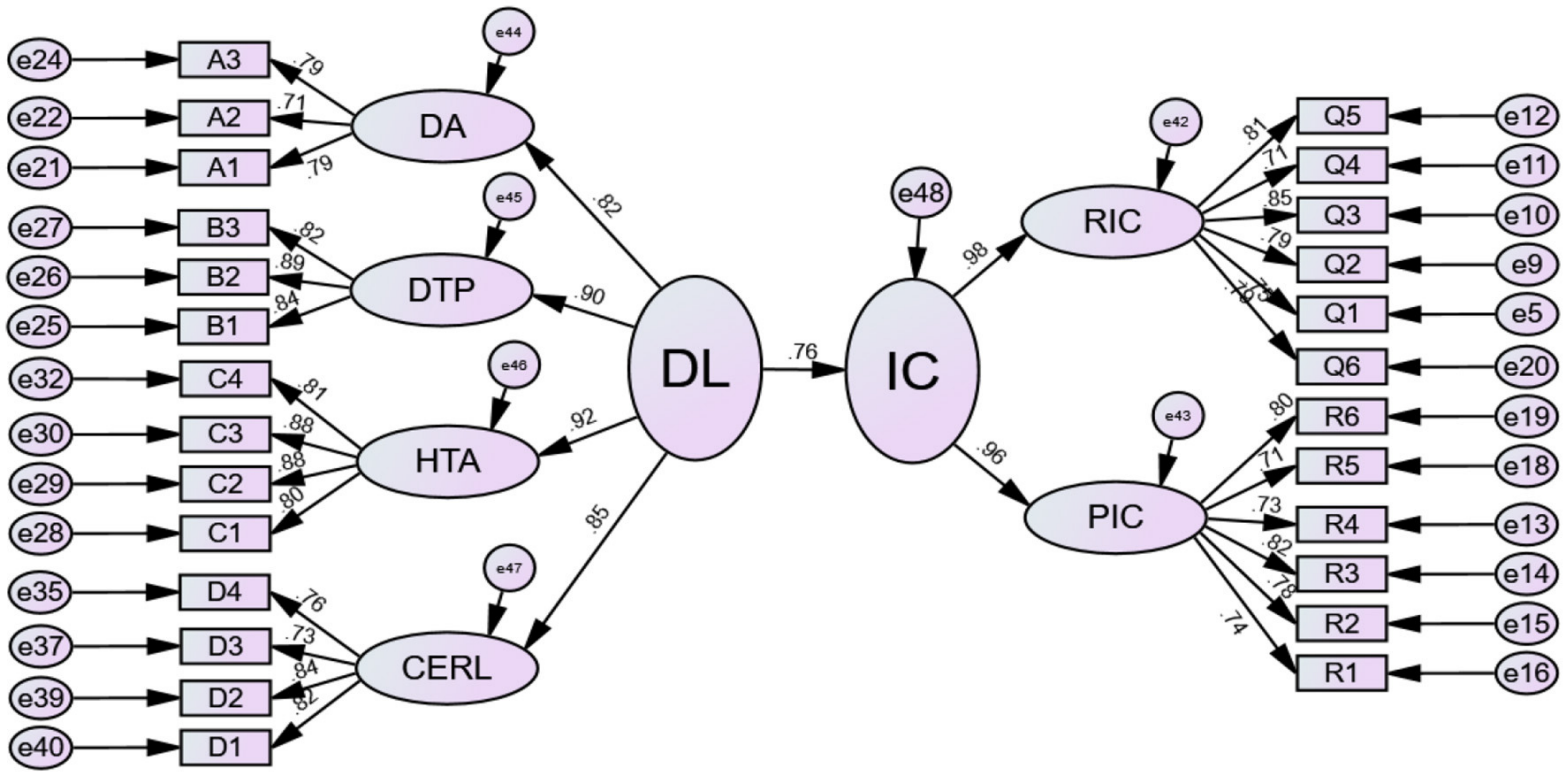
Path diagram of the structural equation modeling analysis. DL, digital literacy; IC, innovation capability; DA, digital awareness; DTP, digital technology practice; HTA, high-level thinking and ability; CERL, cognitive emotion and responsibility literacy; RIC, realistic innovation capability; PIC, potential innovation capability.

After confirming the reliability and validity of all constructs in the model, a structural equation model was constructed using Digital Literacy (DL) as the independent variable and Innovation Capability (IC) as the dependent variable. The model was estimated using maximum likelihood, where the path coefficients (β) in terms of their signs and significance were used as indicators to test the impact of DL on the IC. The results of the structural equation modeling analysis are presented in Figure 1.

Overall, DL has a significant positive effect on IC (β = 0.76, *p* < 0.001). Each 1 unit increase in the digital literacy of university students corresponds to a 0.76 unit increase in their innovation capability. This suggests that improving students' digital literacy can significantly enhance their ability to generate new ideas, effectively apply digital tools, and contribute to collaborative innovation in educational and professional settings. This study also highlighted the standardized factor loadings between the observed variables and their corresponding latent variables. All standardized factor loadings were significant at the 0.001 level (*p* < 0.001) and >0.80, indicating that the selected observed variables effectively reflected the latent variables. Among the DL dimensions, the explanatory power was ranked from highest to lowest as follows: HTA (β = 0.92, *p* < 0.001), DTP (β = 0.90, *p* < 0.001), CERL (β = 0.85, *p* < 0.001), and DA (β = 0.82, *p* < 0.001). These results suggest that while all dimensions contribute to improving innovation, higher-order thinking skills (HTA) and digital technology practices (DTP) are particularly critical in shaping students' innovation capabilities. For example, improving students' critical analysis, problem-solving, and digital tool application skills may significantly improve their innovation performance compared with focusing only on basic digital technology applications. Both RIC (β = 0.98, *p* < 0.001) and PIC (β = 0.96, *p* < 0.001) effectively reflected IC, further confirming that PIC should not be ignored when measuring IC. This finding suggests that institutions, teachers, and students should attach importance to innovation skills that are not yet evident but could be developed through appropriate learning and practice.

### 4.4 Heterogeneity analysis

To explore the factors influencing university students' DL and IC, this study conducted heterogeneity analysis on five aspects: gender, education background, academic discipline, institution type, and training experience, using independent samples *T*-tests and one-way ANOVA. The results are shown in Table 7, indicating significant differences in DL and IC based on education level, discipline, institution type, and training experience. The key findings regarding DL are as follows.

**Table 7:** Heterogeneity analysis of DL and IC.

**Variable**	**Classification**	**DL**		**IC**	
		**M**	**SD**	**M**	**SD**
Gender	Male	3.59	0.66	3.66	0.62
	Female	3.59	0.48	3.63	0.49
	*t*	0.25^NS^		−0.82^NS^	
Education level	Junior college (Age range: 18–22 years)	3.50	0.62	3.55	0.57
	Undergraduate (Age range: 22–25 years)	3.66	0.50	3.71	0.52
	*t*	−5.17^***^		−5.40^***^	
Discipline	Engineering, manufacturing and construction	3.73	0.56	3.78	0.52
	Humanities and arts	3.70	0.52	3.72	0.48
	Science	3.67	0.53	3.71	0.58
	Education	3.62	0.68	3.70	0.56
	Social sciences, business and law	3.56	0.52	3.64	0.55
	Health and welfare	3.51	0.31	3.63	0.40
	Agriculture	3.31	0.71	3.38	0.65
	Others	3.46	0.61	3.50	0.59
	*F*	9.24^***^		8.49^***^	
Type of institution	Flagship university	3.72	0.49	3.80	0.54
	General university	3.66	0.46	3.72	0.49
	Independent college	3.61	0.57	3.65	0.54
	Vocational college	3.49	0.62	3.54	0.57
	*F*	10.63^***^		12.68^***^	
Training experience	Yes	3.73	0.50	3.77	0.56
	No	3.48	0.58	3.55	0.50
	*t*	−8.35^***^		−7.48^***^	

^***^p < 0.001; NS, not significant.

1. No significant difference was observed in DL between different genders of university students (*t* = 0.25). This finding supports previous research showing that males and females have equivalent abilities in DL (Hatlevik and Christophersen, 2013; He and Zhu, 2017; Inamorato dos Santos et al., 2023).
2. Undergraduate students had higher DL (*M* = 3.66, *SD* = 0.50) than junior college students (*M* = 3.50, *SD* = 0.62). This may be because undergraduates' DL is related to their previous experience in digital environments in everyday life (Martzoukou et al., 2020). Undergraduates are more likely to be exposed to digital technologies and tools, providing them with more digital experience.
3. Undergraduates majoring in engineering, manufacturing, and construction (*M* = 3.73, *SD* = 0.56), humanities and arts (*M* = 3.70, *SD* = 0.52), and science (*M* = 3.67, *SD* = 0.53) had higher DL than those studying education, social sciences, business and law, health and welfare, and agriculture. This may be because these fields emphasize problem-solving, information literacy, digital content creation, and other relevant skills (Holm, 2024). For instance, engineering students often use more technological tools and apply them in diverse environments (Margaryan et al., 2011).
4. Students from flagship universities (*M* = 3.72, *SD* = 0.49) have significantly higher DL than those from general colleges (*M* = 3.66, *SD* = 0.46), independent colleges (*M* = 3.61, *SD* = 0.57), and vocational colleges (*M* = 3.49, *SD* = 0.62). There are significant differences in DL among students from different types of institutions (Zhou et al., 2023). This may be due to the fact that flagship universities aim to cultivate top talents and produce top academic achievements in China, thus acting as active promoters of the digital transformation of education. For example, the flagship university that participated in the survey highly values digital reform, vigorously promotes robust network support, effectively integrates physical and digital spaces, creates abundant smart education applications, and forms a distinctive new digital education ecology, which has effectively enhanced students' DL.
5. Students who received digital training or attended digital literacy lectures scored higher on average (*M* = 3.73, *SD* = 0.50) than those who did not (*M* = 3.48, *SD* = 0.58), which is consistent with previous research (Zhao et al., 2021) and supports the recommendation that higher education institutions can help students develop digital skills through appropriate training programs (Nagel et al., 2022; Igbo and Imo, 2020).

Furthermore, the heterogeneity analysis of innovation capability was consistent with the above trends, indicating that male and female students are equally capable of being innovative. Undergraduates (*M* = 3.71, *SD* = 0.52), students majoring in engineering, manufacturing, and construction (*M* = 3.78, *SD* = 0.52), humanities and arts (*M* = 3.72, *SD* = 0.48), and science (*M* = 3.71, *SD* = 0.58), students from flagship universities (*M* = 3.80, *SD* = 0.54), and students with relevant training experience (*M* = 3.77, *SD* = 0.56) all had higher average innovation capability scores. These findings further support the positive relationship between DL and IC.

## 5 Discussion

An in-depth analysis of the statistical data confirmed the important relationship between digital literacy and innovation capability as follows: First, the results of the correlation analysis indicated that cognitive emotion and responsibility literacy (CERL) had a stronger positive correlation with innovation capability (*r* = 0.72–0.73, *p* < 0.001) among the dimensions of digital literacy, while the other three dimensions had relatively weaker positive correlations with innovation capability (*r* = 0.59–0.67, *p* < 0.001). The reason may lie in the fact that CERL encompasses capabilities such as data security, digital ethics, lifelong learning, and sustainable development, which are important drivers for generating disruptive innovation ideas. For example, a mindset of lifelong learning can enhance people's ability to continuously develop and adapt (Kraus et al., 2023), reduce fear of innovation (Polat, 2025), and thus participate in and lead innovative projects more actively. In contrast, we believe that the core issue with weak associations between digital technology practice (DTP) and innovation capability lies in their inherent differences in attributes. DTP is characterized by proficiency in operating specific technical tools, such as software use and data processing skills, essentially applying rules within established frameworks. This characteristic determines that its contribution to innovation is concentrated on efficiency improvements rather than fundamental breakthroughs.

The above correlation analysis results not only provide specific reform directions for higher education policies but also reveal the dynamic evolution characteristics of digital literacy in the era of generative AI. Future research needs to closely integrate with technological development trends, promote the leap from “skill training” to “literacy cultivation” in digital literacy education, and ultimately achieve a comprehensive improvement in innovation capabilities and maximize social value. Specifically, current Chinese higher education digital literacy courses generally focus on technical operations (such as office software, programming basics), and it is necessary to accelerate the addition of digital ethics courses in general education, combining cases of abuse of generative AI to cultivate students' risk identification ability and sense of responsibility. In addition, policy formulation should consider both local characteristics and international integration. For example, China can rely on the “Global MOOC and Online Education Alliance” to promote global collaboration on standards for ethical education in generative AI.

Second, the structural equation model estimation results show that improving digital literacy has a positive effect on university students' innovation capability (β = 0.76, *p* < 0.001). This implies that Chinese higher education institutions should step out of the traditional academic curriculum framework and accelerate the construction of a digital literacy cultivation ecosystem, in order to stimulate students' intrinsic motivation for innovation. For students, given that digital literacy is a comprehensive ability that encompasses technology, cognition, attitude, and emotion (Martin, 2006; Chan et al., 2017; Brown et al., 2020), they should enhance their digital awareness, proactively learn the digital technologies and tools needed for study, work, and life in the digital age, and focus on key qualities and abilities beyond intellectual factors and digital intelligence.

Finally, the heterogeneity analysis revealed that students' digital literacy is influenced by four factors: education level, discipline, type of institution, and training experience, all of which also influence innovation capability, but gender is not one of them. As the world becomes increasingly digitalized, the gap between males and females in their ability to access, use, and benefit from digital technologies, that is, the so-called gender digital divide, has attracted much attention and discussion in higher education (Ancheta-Arrabal et al., 2021; Palomares-Ruiz et al., 2021). However, this study found that there is no apparent gender gap in digital literacy among university students, indicating that both males and females have equal digital literacy skills, which aligns with previous research (Hatlevik and Christophersen, 2013; He and Zhu, 2017; Inamorato dos Santos et al., 2023). This challenges the traditional perception that there is a digital gender gap in digital skills between men and women (OECD, 2018; Siddiq and Scherer, 2019). The phenomenon of gender equality in digital literacy among the samples may be the result of the combined effects of economically developed, open, and inclusive social cultures and the widespread adoption of digital technology. This suggests that through systematic educational reforms and social support, developing countries can break free from the constraints of the traditional gender division of labor and achieve educational equity in the digital age. This implies that higher education institutions need to consider whether they should revise traditional assumptions about gender differences when designing systems for the development of digital literacy based on actual circumstances.

The study found that undergraduate students had higher levels of digital literacy than junior college students. The different orientations of these educational programs contribute to these differences. Vocational education places greater emphasis on practical skills at the expense of scientific principles, whereas undergraduate education, while valuing practical skills, also emphasizes the holistic development of learning, thinking, and research capabilities. Consequently, undergraduate education provides a more systematic framework for professional learning, requiring students to possess higher levels of digital literacy. In addition to the differences in knowledge focus, this also relates to students' previous experiences in digital environments in their daily lives (Martzoukou et al., 2020). Vocational education and training institutions should establish targeted, systematic professional learning frameworks to cultivate digitally literate talent with both theoretical principles and practical skills.

The study also revealed that undergraduates majoring in engineering, manufacturing and construction, humanities and arts, and science exhibit higher levels of digital literacy. The digital literacy situation among students from different majors is complex, making it difficult to draw definitive conclusions. Some scholars believe that graduate and associate degree students in the field of law have the most prominent skills in using the Internet (Owens and Lilly, 2017), while other evidence suggests that students majoring in electronic information engineering have superior digital capabilities compared to those in mechanical engineering and automation (Tao et al., 2025). However, some researchers have found no significant differences between the disciplines (Akçayir et al., 2016). College students from different majors exhibit heterogeneity in digital literacy, which may be related to differences in social and personal factors. Past studies have shown that socioeconomic factors can affect individuals' access to and use opportunities for technological devices during their growth process, potentially shaping their level of digital literacy (Liang et al., 2021). In addition, grade level may also be an important reason behind the differences in digital literacy among college students from different majors, as grade level is linked to demographic variables such as age and educational level. In the future, more social and personal factors could be introduced to further explore their impact on the digital literacy of college students. However, higher education institutions can still use digital technology to analyze the cognitive preferences and learning styles of students from different majors and target improvements in their digital literacy accordingly.

Additionally, there are significant differences in digital literacy among students from different types of institutions; however, overall, the conclusion that undergraduate college students have a higher level of digital literacy than junior college students still holds true. This may be because undergraduate education places more emphasis on interdisciplinary integration and the fusion of advanced technologies. For example, undergraduate programs in science and engineering generally include advanced skill training, such as programming, data analysis, and AI tool application (such as using Python for machine learning practice), while undergraduate programs in the humanities and social sciences gradually introduce modules such as digital humanities and social network analysis. In contrast, vocational education focuses on the quick mastery of job skills, such as office software operations and basic equipment maintenance, lacking the cultivation of algorithmic logic and critical thinking about data. When considering the type of schools, students from flagship universities perform particularly well in terms of digital literacy. As key forces in the transformation of educational technology to digitalization, these universities can establish resource-sharing mechanisms between different universities by leveraging the cross-temporal and spatial characteristics of digital technology, thereby driving the digital transformation of education across organizations.

Finally, students who had previously attended relevant digital training and lectures demonstrated higher levels of digital literacy, which is consistent with previous research (Zhao et al., 2021). In this study, 40% of respondents indicated that they had not received such training or attended lectures, highlighting the need to improve the coverage of digital literacy programs in the region. Therefore, higher education institutions should, on the one hand, provide relevant lectures to help students improve their digital awareness, cognitive emotion and responsibility literacy, and, on the other hand, develop digital literacy training programs integrated with specific professional courses to improve students' ability to apply digital technologies and tools in their studies, life and work.

## 6 Conclusion

This study explored how digital literacy stimulates university students' intrinsic motivation for innovation from a learner's perspective. First, this study reviewed existing frameworks for assessing students' digital literacy and innovation capability, and based on this, developed and implemented a new framework and scale tailored to Chinese university students. Statistical analysis was conducted using a sample of students (*N* = 1,334) from 12 undergraduate and vocational institutions in Ningbo. The study found that improving digital literacy positively affects students' innovation capability (β = 0.76, *p* < 0.001), with a particularly strong relationship between higher-order thinking skills and innovation capability. Additionally, students' personal characteristics (education level, discipline, type of institution, and training experience) are important factors influencing digital literacy and innovation capability. These findings have implications for the development of educational policies to promote innovation and digital literacy in similar institutions.

It must be acknowledged that this study has certain limitations. First, the sample was drawn from one city in Eastern China, and future research should expand the sample diversity to cover more regions. Second, this study only explored the direct effects of digital literacy on innovation capability; future research should explore indirect effects. Third, this study focuses solely on personal characteristics such as gender, education level, discipline, type of institution, and training experience, but does not consider factors such as age, household registration, parental educational background, and personal background before entering university. These questions should be explored in future studies.

## Data Availability

The raw data supporting the conclusions of this article will be made available by the authors, without undue reservation.
