# Supplementary material for: The impact of digital literacy on university students' innovation capability: evidence from Ningbo, China
Source: Front Psychol. 2025 Jul 8;16:1548817. doi: 10.3389/fpsyg.2025.1548817 (PMC12282524; doi:10.3389/fpsyg.2025.1548817)
Supplement: Supplementary file 1 [file Presentation_1.pdf]

Supplementary Material

1. Self-report questionnaire of university students' digital literacy.

Dimensions	Items
Digital Awareness (DA)	1. I am well-versed in the primary digital tools and technologies relevant to my field (e.g., office software, graphic design software, data analysis software, the Internet, big data, virtual reality, artificial intelligence).
	2. I am aware of the impact of digital technologies on reshaping the competitive landscape of the global economy.
	3. I am actively aware of the latest digital tools and technologies pertinent to my major and career.
Digital Technology Practice (DTP)	1. I have a solid grasp of the basic knowledge and methods of information retrieval and can accurately obtain the information I need.
	2. I am proficient creating and processing text, images, videos, etc. using relevant resources.
	3. I am able to utilize digital tools and technologies to create content that meets the requirements of learning, research, and competitions.
Higher-order Thinking and Ability (HTA)	1. I can easily communicate and collaborate with others online to complete tasks.
	2. I can share data, information, and digital content with others through digital tools and technologies.
	3. I can choose and apply appropriate digital tools and technologies to solve problems as needed.
	4. I leverage digital tools and technologies to innovate my learning and lifestyle.
Cognitive Emotion and Responsibility Literacy (CERL)	1. I consciously assess the credibility, reliability, and value of information.
	2. I can effectively deal with the negative effects of digital technologies (e.g., big data killing, reduced concentration, addicted to internet, reduced eyesight, etc).
	3. I comply with internet laws and regulations, and I adhere to proper online conduct.
	4. I can identify gaps in my digital literacy and improve myself in a timely manner.

2. Self-report questionnaire of university students' innovation capability.

Dimensions	Items
Realistic Innovation Capability (RIC)	1. I possess a keen sense of observation and am adept at identifying problems.
	2. I have my own opinions, excel in independent thinking, and avoid blindly following trends.
	3. I am capable of finding new approaches to solving problems when faced with challenges.
	4. I am skilled at utilizing educational resources and tools for self-directed learning.
	5. I enjoy engaging with others and sharing my perspectives.
	6. I excel at solving problems through interdisciplinary and cross-team collaboration.
Potential Innovation Capability (PIC)	1. I am prepared to confront the unknown and the inherent uncertainty that it entails.
	2. I have a strong interest in new developments and am eager to learn and explore them in depth (e.g., ChatGPT, artificial intelligence, autonomous driving).
	3. I undertake proactive steps to acquire skills related to research and competitions (e.g., Python, MATLAB).
	4. I maintain an awareness of the latest developments and trends in my field of study.
	5. I combine my academic expertise with the most recent developments in industry and technology.
	6. The institution offers us with numerous opportunities for research training.
